# Resilient managed competition during pandemics: lessons from the Italian experience during COVID-19

**DOI:** 10.1017/S1744133120000353

**Published:** 2020-09-04

**Authors:** Joan Costa Font, Rosella Levaggi, Gilberto Turati

**Affiliations:** 1 Department of Health Policy, London School of Economics, London, UK; 2 CESIfo and IZA Department of Economics and Management, University of Brescia, Brescia, Italy; 3 Department of Economics and Finance, Università Cattolica del Sacro Cuore – Rome Campus, Rome, Italy

**Keywords:** Covid-19 pandemic, decentralised decisions, governance, health care integration, Italy, managed competition

## Abstract

In the last decades, several European health systems have abandoned their vertically integrated health care in favour of some form of managed competition (MC), either in a centralised or decentralised format. However, during a pandemic, MC may put health systems under additional strain as they are designed to follow some form of ‘organisational self-interest’, and hence face reduced incentives for both provider coordination (e.g. temporary hospital close down, change in the case-mix), and information sharing. We illustrate our argument using evidence for the Covid-19 pandemic outbreak in Italy during March and April 2020, which calls for the development of ‘coordination mechanisms’ at times of a health emergency.

## 1. Introduction

The Covid-19 pandemic has been responsible for around one million deaths worldwide so far, most of which have taken place in hospitals and nursing homes. A significant share of hospital staff has been put in isolation due to contagion in almost every country, and some countries have faced unexpected congestion in their health services. Strikingly, there are also large variations across countries – and even across and within regions in the same country – on the extent of service congestion. The unexpectedly large death toll has opened a discussion on how to organise health care systems to prevent fatalities in the event of a pandemic. The main question is how to improve the decision-making process as concerns access to care and treatment options under a pandemic.

This question is especially important for European health systems that, in the quest to improve ‘value for money’, have replaced vertically integrated models with some form of managed competition (MC) such as the United Kingdom, Spain and Italy to name a few. Under MC, providers compete for patients under a regulatory framework set by public health care purchasers (Carlson *et al*., 2017). The logic behind MC is that, in the absence of large transaction costs, MC allows reaping the benefits from competition in terms of cost reduction (allocative efficiency) and quality enhancement (Jones and Cullis, 1996). The funding of the system still comes from public budgets, but the provision is left to independent providers (public and private), which make decisions on health care provision guided by ‘organisational self-interest’ to attract more patients, and ultimately to improve quality of care. Consistently, the evidence so far suggests that MC delivers efficiency and quality improvements (Gaynor and Town, 2011; Propper, 2018; Siciliani and Straume, 2019) and some patients benefit from having more choice of provider (Le Grand, 2009; Costa-Font and Zigante, 2016). However, while this scheme may work when most care is supplied on an elective base, an important question to investigate is: how does managed competition fare during unexpected pandemic events such as COVID-19?

MC encompasses limited incentives for providers to cooperate. With some exceptions, MC models rely on the assumption that patients' probabilities of needing health care are independent of one another, and that the decision to hospitalise a patient does not reduce the probability of hospitalising other patients (i.e., there are no capacity constraints). Clearly, this assumption does not hold during a pandemic, when hospitals need to cooperate. Lack of coordination might exert serious consequences on both the number of contagions and of deaths, depending on the incentives that different MC systems have in place to enhance cooperation. That is, the governance of MC competition can make a difference in influencing provider coordination at a critical time.

In this paper, we argue that the ‘type’ of MC in place in a health care system can make the difference during a pandemic. More specifically, it influences how agile providers are in responding to coordination needs. Whilst ‘centralised’ (or ‘integrated’) MC formats allow health services (health insurers) to exert control over providers (which is instrumental for swift decision-making), the same is not true under ‘decentralised’ systems. In other words, what in ‘normal’ times performs well, and potentially delivers ‘value for money’, it might backfire during pandemics. In what follows we further elaborate on this point by characterising the two main models of MC. We draw on the evidence of the spread of the disease in Italy and look at its diverse form of hospitals care delivery across regions (Costa-Font and Greer, 2016). More specifically, we study the governance across different regions that have implemented different forms of MC to determine which characteristics can make a difference in the management of a pandemic. A final section concludes and provides policy recommendations.

## 2. Swift response to the pandemic

The management of the Covid-19 pandemic in Europe has become a test for how responsive health care providers are to the needs of the population; more specifically, how resilient is the system of MC in the event of a pandemic. This is especially important when infections take place in hospitals, since delays in providing timely responses give rise to further infections and fatalities. During a pandemic, the probability of needing care is clearly interdependent among individuals; hence, timely health service reaction is the key to reduce the pandemic spread and to treat patients in a safe environment. Some hospitals need to be swiftly transformed into ‘pandemic wards’, while others are left for other cases. If all resources are allocated to ‘Covid hospitals’, it might result in fatal consequences for patients that suffer from other unrelated, but equally serious conditions.

Pandemic wards require a different organisation, even when patients do not need mechanical ventilation. The type of tests along with the availability of treatments required is rather different (Garrafa *et al*., 2020). This is because patients are highly infectious, calling for strict protocols in their management, and patient choice is hampered by insufficient information on the spread of contagion. Furthermore, protective equipment should be available for all staff, to avoid weakening the human resources available to respond to the pandemic. Hence, organisational models based on the competition without an integrated authority may not provide sufficient incentives for a swift response, which can result in a delayed reaction and higher fatalities (Mak *et al*., 2020).

## 3. Models of managed competition

### 3.1 Decentralised managed competition

Unlike centralised MC models, decentralised models require (i) the separation of ‘insurers’ (generally public insurers in most European countries) from providers, (ii) private providers to supply services alongside with public ones, and (iii) standardised set of patient choices (e.g. a catalogue of hospitals available in the area).

The main mechanism driving allocative efficiency is patient choice (typically guided by some form of observed quality dimension), which makes providers react by adjusting their quality to the set prices. In ‘normal times’, the combination of these choices can eventually result in efficient outcomes if a number of conditions are met: (i) information on quality is available, (ii) information is used by patients in their decisions, (iii) public providers have incentives to react to patient choices. The literature clearly emphasises that information and institutional settings are crucial for quality to improve with the degree of competition (Gaynor and Town, 2011; Gutacker *et al*., 2016; Siciliani *et al*., 2017; Dardanoni *et al*., 2018; Moscelli *et al*., 2018). In fact, it is patients ability to exploit the available information on the differences in quality between providers (which makes them choose the best provider) that eventually allows the system to achieve efficient outcomes (Eurostat, 2017; Siciliani *et al*., 2017; Rechel *et al*., 2018; Levaggi and Levaggi, 2020).

However, the performances of MC may be suboptimal during a pandemic event. Health care providers are deemed to make important decisions, in a context where incentives are mainly for them to act following their own organisational self-interest. However, respond to a pandemic, it is efficient for hospitals to stock and make available personal protective equipment (PPE), to collaborate with other hospitals in purchasing decisions, as well as to test staff and to define protocols to deal with infected personnel. However, given that these activities entail extra costs, hospital managers facing MC (especially in less efficient hospitals) are incentivised to limit safety procedures, especially when even scientists are unsure about the clinical consequences of the infection as was clearly (and still is) the case for Covid-19.

Such organisational self-interest can undermine safety, without improving qualiy as patients cannot obtain information on hospital ‘quality’ concerning the risk of being infected. Relevant information may in fact reach patients with a considerable delay, which reduces the effectiveness of actions at the individual level to reduce the contagion. The same logic applies to the purchase of PPE: uncoordinated purchases might incentivise managers to stock PPE for their own hospital rather than sharing them with the most exposed providers. The regulator could of course step in during a pandemic but at the cost of further delay because in a decentralised model it has no direct day-to-day experience with purchasing medical and safety equipment.

### 3.2 Centralised managed competition

A centralised system of MC rests on different premises: (i) not all providers are split from the control of public purchasers (‘insurers’), and (ii) the role of private providers is fairly limited although they are quite well integrated into the network of public providers. In normal times, a centralised system might not face the same incentives for efficiency as a decentralised system (nor the same incentives for the exploitation of potential market failures). In fact, although public providers can compete for patients, integration in a network may reduce the incentives to outperform other hospitals in the network.

In addition, when authority is integrated, health care regulators have a central role in the process of closing down hospitals, of changing their case mix by imposing the opening of ‘Covid wards’ in some, and of purchasing PPE. The coordination among providers and the integration among services (transfers in and out of nursing homes, providing home care) is supervised by the regulator.

Networks under an integrated authority can reduce the risks of local shortages of PPE. They can react promptly to reduce contagion at the hospital level by devoting some facilities to the emergency while leaving other hospitals free to carry on with normal hospital treatments.

## 4. Managed competition in Italy during the Covid-19 pandemic

One of the most illustrative cases of the trade-off between centralised and decentralised MC is Italy. This country was the first one to be severely hit in Europe by the Covid-19 pandemic. To date, the number of official figures has raised to about 276,000 cases and 35,500 deaths. In Italy alone, health staff represented about 12% of all cases according to reports of the Istituto Superiore di Sanità.^1^ The spread of the contagion has been highly concentrated in few Northern regions: 60% of the cases are concentrated in Lombardy, Emilia Romagna and Veneto.

The Italian NHS is regionally decentralised, and regions can make decisions about the organisation of both inpatient and outpatient care. In particular, since 1992, regions can adopt their own model of MC. Yet, although most regions preferred a more centralised model (Emilia Romagna and Veneto), others (Lombardy) opted for more decentralised solutions (Turati, 2013).

Emilia Romagna adopted an MC model based on health care integration, whereby public hospitals are in integrated networks, and the intensity of use of private providers is determined by the regional authority, which sets quality goals for all the providers. Half the hospital beds are under the control of the regional health service (Table 1). Emilia Romagna operates using intermediate purchasing authorities (‘Area Vasta’, literally ‘Extended Area’) between the regional regulator and the providers, which carry out – in addition to health care purchasing – other functions related to logistics, information technology, financial administration and human resources.

**Table 1.** Beds by hospital type and fatalities

Region	Beds under direct control (%)	Beds in autonomous public hospitals (%)	Beds in autonomous private hospitals (%)	Covid deaths %^a^	% of death of infected patients^b^	% of swabs (^b^) per inhabitant (^c^)
EMILIA ROMAGNA	8235 (50%)	4353 (27%)	3716 (23%)	0.423	0.139	0.0410
VENETO	10,643 (67%)	2748 (17%)	2466 (13%)	0.175	0.081	0.0716
LOMBARDIA	–	25,908 (75%)	8565 (25%)	0.858	0.181	0.0375
ITALY	77,593 (41%)	62,208 (33%)	47,628 (26%)	0.264	0.136	0.0328

For the first three columns the source is the Annuario Statistico del SSN.

a Updated at 30 April 2020 Source (Istat and ISS, 2020).

b Updaed at 30 April 2020 Source (Ministero-della- and Salute, 2020).

c Update at 1 January 2019 Source Istat.

Another example of centralised MC is the regional health service in Veneto. Patients can choose between private and public providers, but a centralised authority controls the management of public hospitals. About two-thirds of beds are under the control of the regional health service whether public or private (Table 1). Although in principle hospitals may compete for patients, most of the competition is among public providers. Similar to Emilia Romagna, Veneto has a purchasing agency (the so-called ‘Azienda Zero’), which controls the management and planning of public hospitals, centralises the purchase of medicines and PPE, and carries out functions related to logistics and human resources. This model, at time of a pandemic, limits the potential shortages of PPE, and allows the selective lockdown of hospitals when needed.

Lombardy has instead adopted a more decentralised MC model, based on an unambiguous separation between purchasing and providing functions (Brenna, 2011). Public hospitals have been transformed into autonomous organisations which compete with private providers. The regional health service defines the general health care goals, but it is not directly involved in the supply of care. In 2015, a new reform has enhanced the autonomous role of public hospitals by further separating purchasers (Aziende Tutela Salute, ATS) from providers (Aziende Socio-Sanitarie Territoriali, ASST). Each ASST consists of a public hospital that competes with private providers and a second department that provides (limited) integration between different levels of care.

In the event of a pandemic such as Covid-19, no hospital bed in Lombardy was under the direct control of the regional health authority (Table 1), which had to undergo negotiations and redraft agreements to arrange for the treatment of Covid-19 patients in private hospitals. Lombardy stepped in to purchase PPE and other devices through the renewed central purchasing authority (set in July 2019 to support public purchasers across the Region), but it had to compete with private hospitals. This led to inevitable coordination failures, unnecessary delays in information sharing between hospitals, and an increased risk of infection for hospital staff (Rosenbaum, 2020).

Although a 6-month national emergency was declared on 31 January 2020 (see the Covid-19 Health Systems Response Monitor^2^) it had almost no immediate repercussion on the organisation of health care. This is why the official start of the pandemic in Italy dates to 20 February, when the first patient tested positive for Covid-19, after being admitted to hospital in Codogno (Lombardy).

**Figure 1.**
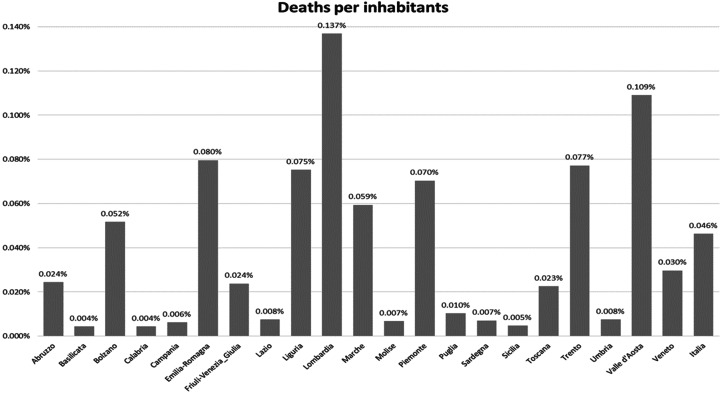
Deaths per at regional level.

Table 1 shows Covid-19 deaths in the three regions under analyisis during the months of March and April 2020 and shows that Lombardy had larger fatalities during the pandemic than other regions (in March and April 80% of deaths are due to Covid-19). Lombardy exhibited the highest ratio of deaths per patients infected and a rather low ratio of swabs per inhabitants. Even when we adjust deaths by inhabitants across all regions in Figure 1, Lombardy appears to be the region with the largest death rate per inhabitant. This result is even most striking when observing that the share of the elderly, the group most hit by the virus, is largely comparable across the three regions. According to official data provided by Istat-Health for all, Lombardy, Veneto and Emilia Romagna are characterised by about 22–23% of over 65 out of the total population in recent years (2016–2018); a similar result is obtained when looking at the share of over 85, 3–4%, across the three regions. These numbers are close to national averages, though the share is lower in the South. Also, the unemployment rate is quite similar, about 6–7%, well below the national average of about 11%.

At the beginning, Lombardy was likely to be initial epicentre. According to a recent study (Cereda *et al*., 2020), the virus was certainly around in Lombardy as early as mid-January and the genetic sequence of the virus that spread in Codogno is different from the one in Bergamo. This means that the Region was hit from several fronts at the same time; and the importance of logistics in the local economy (in the area around Bergamo) may have helped the spread of the virus.

**Figure 2.**
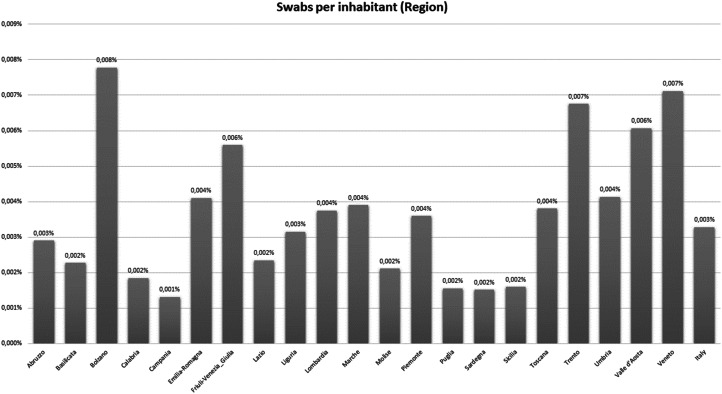
Swabs per inhabitant (at regional level).

Importantly, the testing policy in Italy has followed different regional strategies, which may have triggered an increase (decrease) in the death rate since the denominator (the number of people infected) in some regions may be far higher than the official number as depicted in Figure 2.

This evidence is consistent with the idea that the decentralised model of MC may have exacerbated the effects of the pandemic, measured in terms of fatalities, in Lombardy (Odone *et al*., 2020). Furthermore, quite a few regional health care systems were not able to produce an effective response to the Covid-19 outbreak in nursing homes (Berloto *et al.*, 2020). Nursing homes are independent entities with respect to hospitals, which again makes coordination and information sharing more difficult. Although further statistical analysis might measure the exact extent of ‘excess mortality’, descriptive evidence points to the disadvantage of decentralised models of MC in times of a pandemic.

## 5. Conclusions

The unexpected onset of a pandemic changes the principles and mechanisms that drive the organisation of inpatient care and the design of MC, which is guided by principles of ‘organisational self-interest’. Although MC may play a role in improving the efficiency and quality of care during regular times, during a pandemic event, efficiency calls for a swift response to new needs, and – primarily – information sharing and coordination among health care providers. During the present pandemic, this has led some countries to respond rather differently (Forman *et al*., 2020): some countries have even ‘nationalised’ private health care providers (Ireland and Spain) when there were not enough beds available. And a call to further re-centralisation was advocated also in Italy.

In this article, we have discussed both the principles and empirical evidence that define the organisation of MC in Italy to illustrate the fact that regions that adopted a more centralised model of MC were more resilient, which in turn might have helped to reduce fatalities.

We show that the way in which MC responds to a pandemic largely depends on its design. In highly decentralised systems, the risk of coordination failure is very high, as illustrated in Lombardy. Perhaps the best option available to enhance a swift coordination is to use Pandemic Plans. These should define a mechanism of information sharing in a timely manner and allow some functions to be centralised during the pandemic. In contrast, these plans are less imperative in centralised systems, given that their governance is more amendable to organisational co-operation.

In Italy, a Pandemic Plan was available both at the national as well as at the regional level, but both levels were not prepared for the unexpected rise of Covid-19 patients. The lesson that emerges is that the regional organisation of care determined the initial responses to the crisis, and crucially determined the different outcomes.

Although during good times a more decentralised model of MC might improve health outcomes, during a pandemic a centralised model appears to help to reduce the number of fatalities. In fact, while in normal times, recent empirical evidence suggests that the Lombardy model is more efficient than Emilia Romagna and Veneto (Levaggi *et al*., 2020), in the present pandemic the regional system in Lombardy has underperformed relative to the other two regional health care models. The governance of the Italian health system during the Covid-19 pandemic reveals a trade-off between what ‘works in regular times’ and what is ‘appropriate during a pandemic’.

If pandemics become recurrent, either a centralised form of MC or some specific interventions should be designed to allow a swifter reaction to the need for information and coordination. This carries important lessons for other European countries that have health systems organised according to a decentralised model of MC.

## Footnotes

1 See, e.g., https://www.epicentro.iss.it/coronavirus/bollettino/Bollettino-sorveglianza-integrata-COVID-19_7-luglio-2020.pdf.

2 For information and updates see https://www.covid19healthsystem.org/countries/italy/livinghit.aspx?Section=5.1%20Governance&Type=Section
